# The Ribosome-Binding Mode of Trichothecene Mycotoxins Rationalizes Their Structure—Activity Relationships

**DOI:** 10.3390/ijms22041604

**Published:** 2021-02-05

**Authors:** Weijun Wang, Yan Zhu, Nadine Abraham, Xiu-Zhen Li, Matthew Kimber, Ting Zhou

**Affiliations:** 1 Guelph Research and Development Centre, Agriculture and Agri-Food Canada, Guelph, ON N1G 5C9, Canada; weijun@uoguelph.ca (W.W.); Yan.Zhu@canada.ca (Y.Z.); nabrah02@uoguelph.ca (N.A.); Xiu-Zhen.Li@canada.ca (X.-Z.L.); 2 Department of Molecular and Cellular Biology, University of Guelph, Guelph, ON N1G 2W1, Canada; mkimber@uoguelph.ca

**Keywords:** mycotoxins, trichothecene, mechanism of structure–activity relationships, binding mode, ribosome RNA, binding contacts, binding pocket architecture

## Abstract

Trichothecenes are the most prevalent mycotoxins contaminating cereal grains. Some of them are also considered as the virulence factors of *Fusarium* head blight disease. However, the mechanism behind the structure-activity relationship for trichothecenes remains unexplained. Filling this information gap is a crucial step for developing strategies to manage this large family of mycotoxins in food and feed. Here, we perform an in-depth re-examination of the existing structures of *Saccharomyces cerevisiae* ribosome complexed with three different trichothecenes. Multiple binding interactions between trichothecenes and 25S rRNA, including hydrogen bonds, nonpolar pi stacking interactions and metal ion coordination interactions, are identified as important binding determinants. These interactions are mainly contributed by the key structural elements to the toxicity of trichothecenes, including the oxygen in the 12,13-epoxide ring and a double bond between C^9^ and C^10^. In addition, the C^3^-OH group also participates in binding. The comparison of three trichothecenes binding to the ribosome, along with their binding pocket architecture, suggests that the substitutions at different positions impact trichothecenes binding in two different patterns. Moreover, the binding of trichothecenes induced conformation changes of several nucleotide bases in 25S rRNA. This then provides a structural framework for understanding the structure-activity relationships apparent in trichothecenes. This study will facilitate the development of strategies aimed at detoxifying mycotoxins in food and feed and at improving the resistance of cereal crops to *Fusarium* fungal diseases.

## 1. Introduction

Trichothecenes are a large group of mycotoxins produced by various fungal species, mainly from the genera of *Fusarium, Myrothecium*, *Trichoderma*, *Cephalosponium* and *Verticimonosporium.* The contamination of trichothecenes is widely found in forages and multiple grains, such as maize, wheat, barley, rye and oat, posing a global threat to the health of humans and animals [1,2]. Trichothecenes also contribute to the spread of *Fusarium* head blight (FHB) disease, which significantly decreases the yield and quality for the global production of multiple important grains [3]. Therefore, many efforts have been devoted to developing effective strategies to manage trichothecenes for food/feed safety and security [3,4].

**Figure 1:**
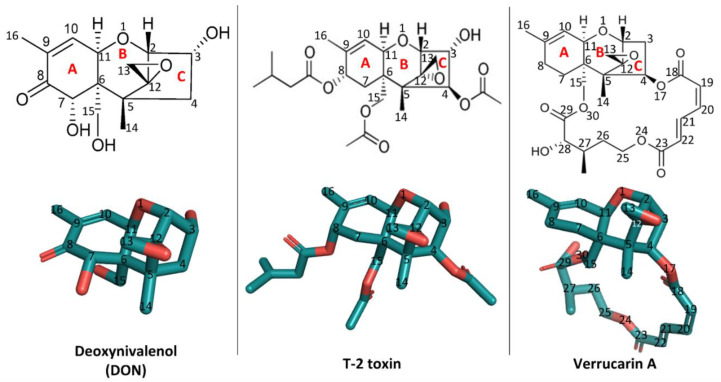
The two-dimensional and three-dimensional structures of deoxynivalenol (DON), T-2 toxin, and verrucarin A and their position numbering. The three-dimensional structures were visualized using PyMOL 2.3.4. The PDB accession number of DON is 3J6, those of T-2 toxin and verrucarin A are 3 L2 and ZBA, respectively.

Structurally, trichothecenes are a large family of sesquiterpenoid mycotoxins that share a core structure of tricyclic 12,13-epoxytrichothec-9-ene (trichothecene) ring but differ in the substituents at various positions (Figure 1). Based on the substitution pattern, trichothecenes can be subcategorized into four major groups, namely type A, B, C, and D, with currently over 150 trichothecenes identified [1]. Among them, the trichothecenes of highest concern include deoxynivalenol (DON, type B), nivalenol (NIV, type B), diacetoxyscirpenol (DAS, type B), T-2 toxin (type A) and their derivatives, due to the prevalence of their contamination in food and feed [4]. The structure–activity relationship of trichothecenes has been extensively researched [5]. Specifically, the C^12,13^-epoxy ring, the double bond between C^9^ and C^10^, and C^3^-OH in type A and B trichothecenes have been identified as key structural elements contributing to their toxicity (Figure 1) [2,4,5,6]. Microbial metabolism plays an important role in modifying these key structural elements, thus altering the toxicity [4,7]. For example, in ruminants, DON and NIV undergo a reductive de-epoxidation of the C^12,13^ epoxide ring to form de-epoxy metabolites, which display reduced cytotoxicity [8]. This transformation, attributed to anaerobic gut microflora, motivated the search for microbes capable of de-epoxidation for detoxification. The C^3^-OH epimerization is another microbial transformation pathway identified for DON detoxification [9,10,11]. Among trichothecene mycotoxins, substituent groups attached to the trichothecene ring system modulate their toxicity in different patterns [5]. For example, in comparison with DON, T-2 toxin has two additional acetyl groups (-OCOCH_3_, short as Ac) at C^4^ and C^15^ positions and one additional isovaleryl group (-OCOCH_2_CH(CH_3_)_2_) at the C^8^ position, all linked through ester bonds but lacks C^7^-OH (Figure 1). These substitutions conferred T-2 toxin higher toxicity than DON [5]. In contrast, the substitution at C^3^-OH position by a glucosyl group reduced intestinal toxicity and phytotoxicity of deoxynivalenol 3-glucoside (DON-3-Glc) relative to the DON [12,13]. Besides glycosylation, in-planta metabolism of trichothecenes also involves conjugation of polar molecules, including amino acids or glutathione (GSH), to the C^3^-OH group [14]. Although a promising means for detoxification, these conjugates may be hydrolyzed back to their parent mycotoxins in the digestive tract of mammals and, for this reason, are often referred to as masked mycotoxins. In mammals, trichothecene metabolism also involves biotransformation events by the aforementioned gut microflora as well as phase II xenobiotic detoxification involving glucuronidation at the C^3^-OH position [7,15,16]. Altogether, why and how these key structural elements, substituent groups, and biotransformed metabolites impact the toxic actions of trichothecenes has not been systematically rationalized. Filling this knowledge gap in the mechanisms behind the structure–activity relationship of trichothecenes is critical for developing strategies to manage this large family of mycotoxins.

The toxicity of trichothecenes is a long-standing research topic. In mammals, trichothecenes displayed various toxic effects, including growth retardation, feed refusal, vomiting, immunosuppression, and reproductive disorders [2,4,17]. To plants, trichothecenes also cause growth defects such as inhibition of root elongation and dwarfism of seedlings and bleaching of leaves and other tissues [6,18,19]. These toxic effects at the organism level are manifestations of the cytotoxicity at cellular and molecular levels. Multiple cellular and molecular impacts have been observed for trichothecenes, including the inhibition of eukaryotic protein synthesis, inhibition of cell division and RNA/DNA synthesis, activation of mitogen-activated protein kinases (MAPKs) signaling pathways, alteration of membrane integrity, disruption of mitochondria function, induction of cytokine gene expression and cell apoptosis, and others [5,6]. Among these, the inhibition to the eukaryotic protein synthesis is the primary mode of toxic action of trichothecenes [20,21]; other observed cellular effects likely reflect secondary effects of protein synthesis inhibition [6].

Trichothecene mycotoxins bind non-covalently to the peptidyl transferase site of ribosomes, leading to protein synthesis inhibition [21,22]. A key insight into the mechanism of inhibition comes from a broad study investigating the binding mode of diverse toxins to the 60S large subunit of *Saccharomyces cerevisiae* ribosome by X-ray crystallography. In this study, the trichothecenes of DON, T-2-toxin, and verrucarin A were found to bind at the A-site of the peptidyl transferase center in the 60S subunit, and were therefore classified as A-site inhibitors together with nagilactone C, anisomycin, and several plant alkaloids (e.g., lycorine, narcilasine and homoharringtonine). This breakthrough provided guidance for subsequent structure-based drug designs [23]. While an important study [23], the broad focus precluded the authors from deeply discussing the structural details of the trichothecene binding mode to the yeast ribosome. Afterward, Oswald’s research group analyzed the yeast 80S ribosome structure complexed with trichothecenes and preliminarily reported three hydrogen bonds contributed by C^3^-OH, oxygen in the C^12,13^-epoxide ring, and C^15^-OH of DON, respectively [12,24,25]. Dellafior et al. (2017) investigated the structural basis underlying the ribotoxic activity of trichothecenes using docking simulations and pharmacophoric analysis [26,27]. Altogether, this structural information opens a new window to understand the structure–activity relationship of trichothecenes.

The present work aims to understand the mechanism behind the structure-activity relationship of trichothecene mycotoxins. Herein, we took an in-depth and comparative examination of the existing yeast 80S ribosome structure complexed with T-2 toxin, DON, and verrucarin A, which represent type A, B, and D trichothecenes, respectively [23]. The architecture of the binding pocket and these binding interactions explain why and how these key structural elements and substituents in trichothecenes specifically impact their toxic actions. Therefore, the present study elucidates the mechanism behind the structure–activity relationship for trichothecenes. It will further facilitate the strategy development for mitigating this large group of mycotoxins in food and feed, as well as to improve the resistance of cereal crops to *Fusarium* fungal diseases.

## 2. Results

Trichothecenes displayed a strong inhibition to the yeast growth. As a simple and popular eukaryotic model organism in biological research, wild type or engineered *S. cerevisiae* strains have been used to investigate the molecular mechanism of toxic action of trichothecenes, leading to the identification of several genes associated with resistance to trichothecenes [13,28,29,30]. In addition, breakthroughs in solving x-ray crystal structures of *S. cerevisiae* ribosomes complexed with trichothecenes [23] enables us to further investigate the interactions between the large ribosome subunit and these ligands at the atomic level. In analyzing these currently available X-ray structures using PyMOL, it is important to keep in mind the limitations of these structures (PDB accession number: 4U6F, 4U53, and 4U50). In particular, ribosomes are large entities and diffract to limited resolution. The structures discussed here were refined to between 3.1 and 3.3 Å resolution data. Note that the phosphate groups in RNA and abundant metal ions dominate ribosome scattering, and individual lighter carbon atoms contribute little to high-angle scattering; the effective resolution of the toxins is lower than the RNA. Helping mitigate this is the observation that the A-site is in the most ordered, and therefore the best-resolved region of the structure in these crystals. At this resolution, the orientation of small, flexible groups can be difficult to model reliably and may occupy slightly different positions in the two copies in the structure. In addition, light elements (e.g., N, C, and O) cannot be reliably distinguished, and solvent atoms are not modeled; this is important as these toxins likely make additional indirect, water-mediated hydrogen bonds to nearby bases, but these are not directly visualized in the structures. It is worth noting that the peptidyl transferase center (PTC) is a highly conserved ribosome region across Archaea, Bacteria, and Eukarya domains, thus allowing us to extrapolate present results to other eukaryotic organisms from plant, fungi, and mammals [31,32].

### 2.1. The Multiple Interactions Involved in the Deoxynivalenol (DON) Binding to Yeast 80S Ribosome

**Figure 2:**
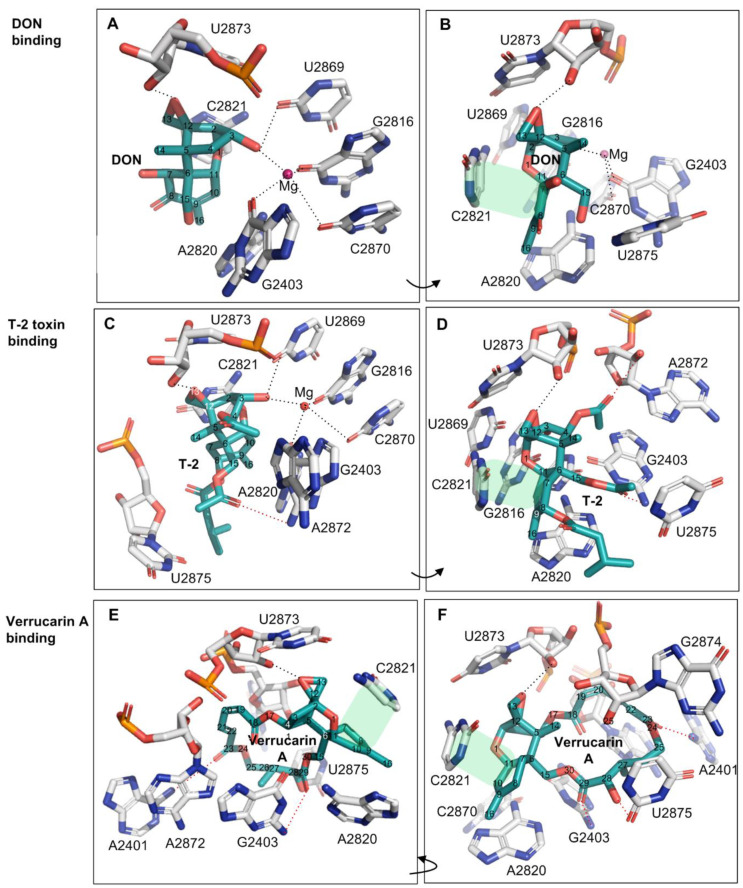
The binding of trichothecene mycotoxins of DON, T-2 toxin and verrucarin A to yeast 80 S ribosome. (**A**,**B**): The binding contacts of DON to 25S rRNA; (**C**,**D**): The binding contacts of T-2 toxin to 25S rRNA; (**E**,**F**): The binding contacts of verrucarin A to 25S rRNA. The hydrophobic stacking interaction between two rings was shaded in light green, and the additional contacts relative to DON were labeled in the red dash lines.

Deoxynivalenol (DON) is the most prevalent type B trichothecene mycotoxin in grains with a relatively simple substitution structure. In the crystal structure of DON binding to the yeast 80S ribosome (PDB accession number: 4U53), the ligand DON is anchored in a binding pocket formed exclusively by 25S rRNA, a component of the large subunit (60S) of the ribosome (80S) (Figure 2A,B). Specifically, the C^3^-OH of DON acts as a ligand (the C^3^-OH oxygen is 2.7–2.9 Å from Mg^2+^), coordinating the Mg^2+^ ion also bound by O^2^-C2870, O^6^-G2816, and O^6^-G2403. This group also forms one hydrogen bond as a donor with O^2^ of G2869 (2.7–3.2 Å) (Figure 2A and Table 1). As a key toxicity-contributing element, the oxygen in the C^12,13^-epoxide ring hydrogen bonds with the -HO^2′^ group in the β-ribose of U2873 (2.5–2.8 Å) (Figure 2B, Table 1). Open space adjacent to this group implies a possible second hydrogen bond to a structured water group. Notably, three of these contacts have been briefly mentioned by Pierron et al. (2016), but the specific atoms participating in the interactions were not identified [24] (Table 1, the contacts labeled with *). The C^15^-OH, C^7^-OH, and C^8^-O line one side of DON and are solvent-exposed; the groups do not make direct hydrogen bonds to 25S rRNA, but instead are likely to hydrogen bond to ordered water molecules in the binding site. Other than the above polar interactions, DON binding is also stabilized through multiple apolar interactions, including hydrophobic stacking interactions between the six-membered ring of C^6–11^ in DON (ring A) and the cytosine ring of C2821 (Figure 1). In addition, the C^9^ = C^10^ group in bond in DON (along with the C^16^ methyl) stacks in a wedge-shaped pocket formed between a pair of successive bases—the cytosine ring of C2821 and the adenine ring of A2820. Overall, DON fits tightly in its pocket, with extended van der Waals contacts, and is also highly pre-organized to sit in this pocket. In summary, the oxygen in the C^12,13^-epoxide ring, the double bond between C^9^ and C^10^, and C^3^-OH contribute key interaction contacts for the DON binding to the 25SrRNA in the ribosome. Consistent with this view, the C^12,13^-epoxide, C^9^ = C^10^ double bond and C^3^-OH in type A and B trichothecenes have been previously reported as the key toxicity-contributing elements in DON structure [4,5].

### 2.2. The Substitutions of T-2 Toxin and Verrucarin A Introduced the New Contacts for Their Binding to the Ribosome

T-2 toxin represents the most common type A trichothecene mycotoxin with high toxicity. Relative to DON, T-2-toxin has two additional acetyl substituents (-OCOCH_3_, abbreviated as OAc) at C^4^ and C^15^ positions, and one additional isovaleryl group (-OCOCH_2_CH(CH_3_)_2_) at C^8^ position all linked through ester bonds, but lacks hydroxyl group at the C^7^ position. The structure of the T-2 toxin bound to the ribosome (PDB accession number: 4U6F) overlays closely with the common core of the DON structure and makes similar contacts from the common backbone structure. The extended substituents at C^4^, C^8,^ and C^15^ positions of T-2 toxin introduce the additional binding contacts relative to DON. For example, a hydrogen bond between O^1^ of the C^15^-OAc group and HN^2^ of G2403 (3.4 Å) can be found (Figure 2C,D, and Table 1). The acetyl substituent at the C^4^ position (C^4^-OAc) is likely incorrectly modeled: the methyl group makes more chemical sense in the modeled position of the acetyl oxygen (sitting in a nonpolar pocket between G2403 and A2872), while the keto oxygen in the position of the methyl is positioned to make hydrogen bond to A2872 ribose 2′OH (Figure 2C,D and Table 1). In addition, the -isovaleryl substituent at the C^8^ position of T-2-toxin makes extended nonpolar contacts with A2820 and U2875, while the O^1^ oxygen in this substituent is likely hydrogen bonding with structured water molecules (Figure 2D and Table 1). These substituents on T-2, in general, seem to primarily extend the nonpolar contact surface with the ribosome while maintaining a tight structural complementarity. Consistently, T-2 toxin displayed higher toxicity than DON by 43~500 times with the significantly lower relative IC_50_ values between 0.002 and 0.023 (Table 2) when using human and mouse cell lines as the test models [5,33,34].

Verrucarin A is classified as a type D trichothecene and differs from types A, B and C by containing an additional ring linking the C^4^ and C^15^ positions. In comparison with DON and T-2 toxin, verrucarin A lacks the C^3^-OH and thus neither coordinates magnesium ion nor forms a hydrogen bond from C^3^-OH (Figure 2, Table 1). However, the additional ring linked to C^4^ and C^15^ created three new hydrogen bonds (with A2401, G2403, and U2875) while forming extended nonpolar contacts with the exposed ribose bases of A2872, U2873, and G2874 (Figure 2E,F and Table 1). In keeping with these extended contacts, the cytotoxicity of verrucarin A towards Vero cells was reported higher than that of DON by 125~250 times (relative IC_50_ = 0.004~0.008, Table 2) [35].

**Table 2:** The comparison in the substitution type and toxicity of several selected trichothecenes and their derivatives.

Trichothecenes	Substitution Positions and Types						Substitution Type ^a^	Cytotoxicity		References
	C^3^	C^12,13^	C^4^	C^7^	C^8^	C^15^		Test Model	Relative IC_50_^b^	
	Positions for Type II Substitution		Positions for Type I Substitution (C^4^, C^7^, C^8^, C^15^)							
DON	-OH	Epoxide	-H	-OH	=O	-OH	As a reference	-	1.0	
T-2	-OH	Epoxide	-OAc	-H	-OCOCH_2_ CH (CH_3_)_2_	-OAc	Type I	3T3, Hep-G2, A549, HEp-2, Caco-2, A204, U937, Jurkat, RPMI8226, HUVEC	0.002–0.023	[33,34]
HT-2	-OH	Epoxide	-OH	-H	-OCOCH_2_ CH (CH_3_)_2_	-OAc	Type I	3T3, Hep-G2, A549, HEp-2, Caco-2, A204, U937, Jurkat, RPMI8226, HUVEC	0.011–0.046	[33,34]
DAS	-OH	Epoxide	-OAc	-H	-H	-OAc	Type I	SF-9 insect cell	0.011	[36]
15-Ac-DON	-OH	Epoxide	-H	-OH	=O	-OAc	Type I	3T3, Caco-2	1.0–1.1	[8,37]
Verrucarin A	-H	Epoxide	-OR	-H	-H	-OR	Type I	Vero, rat spleen lymphocytes	0.004–0.008	[35]
NIV	-OH	Epoxide	-OH	-OH	=O	-OH	Type I	3T3	0.79	[8]
3-Ac-DON	-OAc	Epoxide	-H	-OH	=O	-OH	Type II	3T3, Caco-2	2.1–10	[8,37]
DON-3-GlcAc	-OGlcAc	Epoxide	-H	-OH	=O	-OH	Type II	K562	>206	[38]
DON-GSH	-OH	-GSH	-H	-OH	=O	-OH	Type II	n.d. ^c^	n.d.	[30]
3-*epi*-DON	-OH	Epoxide	-H	-OH	=O	-OH	NA ^d^	3T3, Caco-2	357–1181	[39]
3-keto-DON	=O	Epoxide	-H	-OH	=O	-OH	NA	3T3, Caco-2	3.0–4.5	[39]
DOM-1	-OH	Epoxide	-H	-OH	=O	-OAc	NA	3T3, chicken lymphocytes	55–517	[8,40]

^a^: DON was used as a reference trichothecene due to its relatively simple structure; type I: substituents either do not impact binding or introduced additional binding contacts; type II: substituents resulted in the steric hindrance for binding and impaired the corresponding binding contacts. ^b^: IC_50_ (the half-maximal inhibitory concentration) values relative to DON. ^c^: n.d. means no data available. ^d^: not applicable.

### 2.3. The Architecture of Trichothecenes Binding Pocket in Ribosome Impacts the Accommodation of Trichothecenes in Two Different Patterns

**Figure 3:**
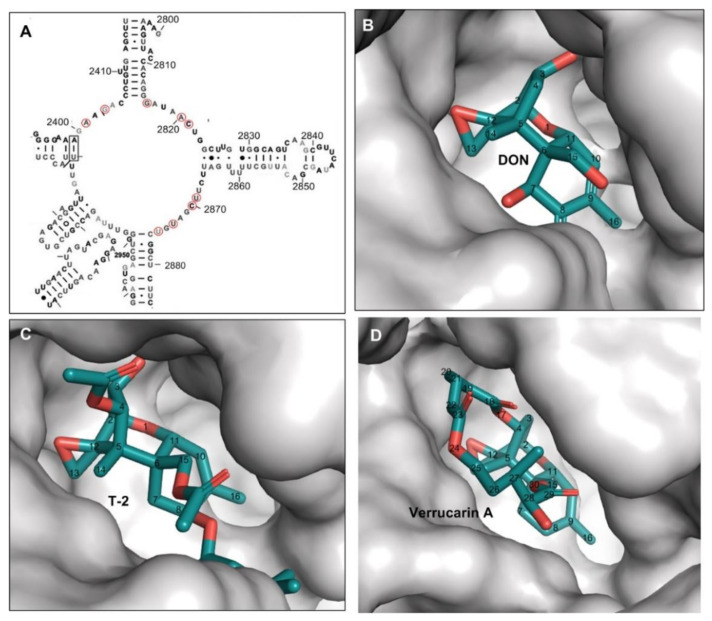
The binding pocket formed merely by 25S rRNA for DON, T-2 toxin and verrucarin A. (**A**) The partial secondary structure of *Saccharomyces cerevisiae* 25S rRNA adopted from the previous report [41], the nucleotides contributing to binding pocket formation were circled in red; (**B**) DON anchoring in the binding pocket; (**C**) T-2 toxin anchoring in the binding pocket; (**D**) verrucarin A anchoring in the binding pocket.

The binding pocket for three trichothecenes (DON, T-2-toxin, and verrucarin A) is formed by the nucleobases from unpaired loops in the 25S rRNA of 80S ribosome, including A2401, G2403, G2816, A2820, C2821, U2869, C2870, U2873, and U2875 (Figure 2 and Figure 3A). The mycotoxin ligands of DON, T-2-toxin, and verrucarin A bind deeply in this pocket (Figure 3B–D). Within this pocket, the oxygen from C^12,13^ epoxide ring, the double bond between C^9^ and C^10^ in ring A and C^3^-OH from T-2 toxin or DON were tightly contacted with the surface wall of the binding pocket through hydrogen bonds or metal ion coordination as described above (Figure 3B–D). This region of the molecule fits tightly in a narrow pocket, and any bulky substitution in these positions will cause significant steric hindrance and likely abolish trichothecenes binding within the ribosome (Figure 2 and Figure 3, Table 1). This type of substitutions is exemplified by the DON conjugates such as deoxynivalenol 3-glucoside (DON-3-Glc) with C3-glucosyl substitution and DON-GSH with C^12/13^ glutathione-substitution. In contrast, the positions of C^16^, C^15^, C^8^, C^7,^ and C^4^ in trichothecenes face into a narrow but extended cleft, which allows substituents to be much more readily accommodated at these positions, and indeed, make additional contacts that may further enhance binding. The example trichothecenes with this type of substitution include T-2 toxin with the acetyl group at C^4^ and C^15^ positions and -OCOCH_2_CH(CH_3_)_2_ group at the C^8^ position, as well as verrucarin A with a linking ring between C^4^ and C^15^. The tolerance of trichothecenes to substitutions is therefore dictated by the architecture of the binding pocket formed by 25S rRNA.

### 2.4. The Binding of Trichothecene Induced the Conformation Change of Several Nucleobases in 25S rRNA

**Figure 4:**
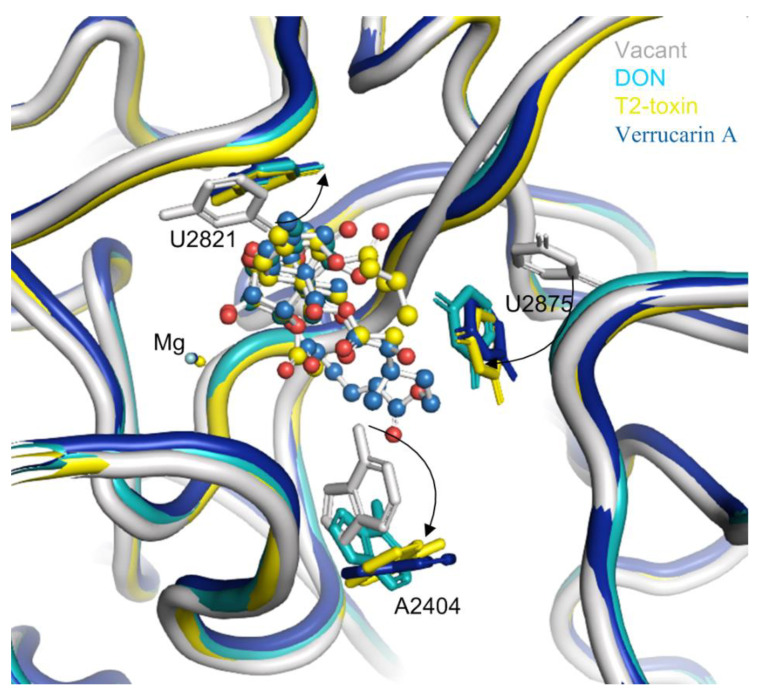
The conformation changes of several nucleobases around the binding pocket in 25S rRNA upon trichothecenes binding. The vacant 25S rRNA was taken as the reference and shown in gray, the 25S rRNA bound with DON was in light blue, the 25S rRNA bound with T-2 toxin was in yellow, and that bound with verrucarin A was in deep blue.

The binding of trichothecenes (e.g., DON, T-2 and verrucarin A) to the yeast ribosome induces conformation changes in several nucleobases in 25S rRNA (Figure 4). Specifically, the uracil ring of U2821 shifted towards the C^6,7,8,9,10,11^ ring (ring A) of trichothecenes to improve the hydrophobic stacking interaction between these two rings. Another uracil ring of U2875 also moved towards ring A of trichothecenes and the substituent of OCOCH_2_CH(CH_3_)_2_ at the C^8^ position of T-2-toxin. This conformational change of the uracil ring in U2875 was also found when anisomycin binding to the 25S rRNA [23]. In addition, a rotation of the adenine-ring in A2404 was observed upon the trichothecenes binding, which is likely to provide additional space for the accommodation of C^15^-OAc substituent in T-2 toxin or the connecting ring between C^4^ and C^15^ in verrucarin A. In contrast, the conformation changes are relatively small for these nucleobases interacting with the oxygen in C^12,13^-epoxide and C^3^-OH in DON and T-2-toxin. These nucleobases included U2873, U2869 and G2816, forming a rigid wall for the partial binding pocket along with divalent metal ion of Mg^2+^. Altogether, these conformational changes of the nucleobases from 25S rRNA lead to a favorable binding pocket architecture for the trichothecenes anchoring to the large subunit of eukaryotic 80S ribosome.

## 3. Discussion

### 3.1. The Key Structural Elements Contributing to Trichothecenes Toxicity Play Critical Roles in the Binding to the Eukaryotic Ribosome

The binding of trichothecenes to the peptidyl transferase site of the eukaryotic ribosome leads to the inhibition of protein synthesis; this is the primary mode of toxic action of trichothecenes [21,22]. Within the chemical structure of trichothecenes, the C^12,13^-epoxide ring, double bond between C^9^ and C^10^ and C^3^-OH in type A and B trichothecenes have been identified as the important structural elements contributing to their toxicity [2,4,5,6]. Our reanalysis of previously published trichothecene 60S ribosome particle complexes helps explicate the critical roles of these key structural elements in contributing the binding contacts for trichothecenes binding to the eukaryotic ribosome (Figure 2 and Table 1). These key binding contacts include hydrogen bonds, metal ion coordination, extended van der Waals interactions, and the burial of nonpolar surfaces, rationalizing the observed functionality of these key structural elements in the toxic action of trichothecenes.

### 3.2. The Substitution Patterns of Trichothecenes Modulates the Binding to the Ribosome and Further Influence Their Toxicity

Trichothecenes share a core structure of tricyclic 12,13-epoxytrichothec-9-ene (trichothecene) ring but differ in the substituents at various positions. These various substituents lead to the structural diversity of trichothecene mycotoxins and confer the different toxicities to these trichothecenes [5]. However, the mechanism of how and why these substitution structures of trichothecenes affect their toxic action differently is largely unknown. Through the comparison of three trichothecenes binding to the ribosome along with the examination of their binding pocket architecture in this study, it was indicated that the substitution could impact trichothecenes’ binding to the ribosome in two completely different ways—enhancing binding or reducing binding. Therefore, we classified them into type I and type II substitution as follows (Table 2).

Specifically, the type I substitutions usually occur at C^4^, C^7^, C^8^, C^15,^ or C^16^ positions in trichothecenes (Figure 3); these groups face into an open (though narrow) cleft, allowing extended substituents to be accommodated without introducing steric clashes. Therefore, these type I substitutions either do not impact trichothecenes binding or introduce additional binding interactions that enhance binding relative to their parent trichothecenes. Ultimately, the enhanced binding affinity to the ribosome will lead to higher toxicity. The group of trichothecenes with this type I substitution are exemplified by T-2 toxin, HT-2, DAS, and others, whose toxicity was found higher than that of their parent trichothecene (e.g., DON) in multiple test models (Relative IC_50_ = 0.002–0.023 for T-2 toxin, 0.011–0.046 for HT-2, and 0.011 for DAS, respectively, Table 2). This concept was further supported by many studies with the type A trichothecenes to investigate the effect of these type I substitutions on its toxicity [5]. For example, the removal of C^4^-acetyl and/or C^8^-isovaleryloxy groups from T-2 toxin resulted in a reduced inhibition to protein synthesis in Vero cells and toxicity [5,35]. On the other hand, the esterification at C^4^ and C^15^ in the backbone of trichothecenes generally increased toxicity towards *Arabidopsis thaliana* leaves [42], as these substitutions generally introduced additional binding contacts between eukaryotic ribosome and trichothecenes and ultimately enhanced their binding affinity (depending on the structure of substitutions).

In contrast, type II substitution often leads to the steric hindrance of trichothecenes binding to ribosomes. These substituents are usually placed at the C^3^, C^12,^ or C^13^ positions, where the key toxicity-contributing groups of trichothecenes make important favorable contacts. More importantly, these groups fit closely within a constricted pocket, and there is no additional space to accommodate substitutions that add additional bulk (Table 2). Therefore, the bulky type II substitution will destroy strongly impair the binding of trichothecenes to the ribosome. This impaired binding affinity will further lead to the reduced toxicity of the corresponding trichothecene relative to their parent molecule (e.g., DON). The trichothecenes with type II substitution are well exemplified by several DON/T-2 conjugates, such as DON-3-Glc, 3-Ac-DON, and 3-Ac-T-2, with the substitutions as C^3^ position, and DON-GSH with glutathione substitution at C^12/13^ position (Table 2). Specifically, from a structural aspect, the type II substitutions at C^3^-OH (e.g., DON-3-Glc, DON-3-Ac) will spatially clash with the bound metal ion (Mg^2+^) and the nucleotides of G2816, U2869 and C2870, and lose the corresponding contacts from the DON-C^3^-OH group to nucleobases and metal ion. Similarly, the glutathione group (-GSH) at either C^12^ or C^13^ position in DON-GSH will spatially conflict with the nucleotides of U2873 and fail to form the corresponding hydrogen bond as seen for DON. Although many other factors influence the toxicity (e.g., absorption, metabolism, excretion, exposure route in the test model, genetics and life stage of test species, and others), the impaired binding to ribosome caused by type II substitutions will generally lead to reduced toxicity of trichothecenes in comparison with their parent mycotoxins (Table 2). For example, the acetyl substitution at the C^3^ position of T-2 (end product: 3- Ac-T-2 toxin) and DON (3-Ac-DON) led to a significant decrease in the inhibition of protein synthesis in Vero cells and rat spleen lymphocytes [35]. The cytotoxicity of the acetyl-T-2 toxin on mouse lymphoma cells was reduced as well [33]. As for the type II substitution at C^12/13^ position, the toxicity of DON glutathione conjugates of DON-GSH has not been characterized to date. However, an FHB resistance gene *Fhb7* was identified recently, which encodes a glutathione S-transferase and confers broad resistance to *Fusarium* species by detoxifying trichothecenes into DON-13-GSH through de-epoxidation [30], indicating reduced phytotoxicity of DON-13-GSH relative to DON.

The correlation between trichothecenes’ toxicity and their potential binding affinity to ribosome modulated by substitutions can be found as discussed above; it is worth noting that many factors may also contribute to the toxicity change of various trichothecenes. For example, lipophilicity is one of the toxicity-influencing factors affecting compound’s absorption, distribution, penetration across membranes, metabolism, and excretion in the cellular test models. Specific substitution may impact trichothecenes’ lipophilicity, and in turn, their toxicity. Intriguingly, the same substituent of acetyl (OAc) at C^3^-OH or C^15^-OH led to a different impact on their toxicity, where 15-Ac-DON displayed similar toxicity to DON (the relative IC_50_ of 15-Ac-DON = 1.0~1.1) while 3-Ac-DON displayed reduced toxicity by 2.1~10-fold relative to DON (the relative IC50 of 3-Ac-DON = 2.1~10) (Table 2). This difference can be rationalized by the substitution patterns of trichothecenes and their binding mode to the ribosome, as discussed above.

### 3.3. Implications of the Mechanism Behind Structure–Activity Relationships on the Development of Detoxification Strategies for Trichothecenes

As discussed above, the present study explained why and how the key structural elements and substituents of trichothecenes impact their toxic actions through the binding interactions with the eukaryotic ribosome. Interestingly, these key toxicity-contributing structural elements have been targeted to develop detoxification strategies towards trichothecenes [43], such as the reductive de-epoxidation at C^12,13^ epoxide ring [44,45,46], and the epimerization at C^3^-OH [10,11,47]. The epimerization and de-epoxidation are two microbial biotransformation pathways reported for DON detoxification with 3-*epi*-DON and deepoxy-DON (DOM-1) as the metabolized products. The 3-*epi*-DON, intermediate 3-keto-DON, and deepoxy-DON (DOM-1) all displayed reduced toxicity relative to DON [24,39]. Microbes responsible for de-epoxidation have been isolated from animal gut microflora and soil environments [4,44,45,48]. For example, under anaerobic conditions, bacterial consortia such as *Eubacterium* sp. BBSH 797 and single isolates such as *Bacillus* sp. strain LS100 detoxify trichothecenes such as DON at a 100% efficiency to DOM-1 [49,50]. In addition, DON is also transformed at a 100% efficiency via the epimerization pathway by *Devosia mutans* 17–2-E-8, a gram-negative soil bacterium. Epimerization proceeds via a two-step detoxification process involving oxidation at the C^3^-OH position to the intermediate 3-keto-DON and stereospecific reduction to 3-epi-DON, which displays reduced toxicity relative to DON [9,10,11]. Detoxification comprises an important part of the stress response mechanism of *Devosia mutans* 17-2-E-8 and involves the upregulation of ATP transporters, ribosome-associated translation inhibitors, and oxidoreductases [9]. Consequently, detoxification occurs as a consequence of co-metabolism since *D. mutans* 17–2-E-8 does not require DON as a sole carbon source but detoxifies it in a range of nutrient media, with the highest transformation observed in corn steep liquor [11]. In contrast to this, certain Gram-positive strains such as *Nocardiodes* WSN05-2 utilize DON as sole carbon sources [51]. *Nocardiodes* WSN05-2 may produce 3-epi-DON as an intermediate, and epimerization may be coupled to another downstream metabolic process.

**Figure 5:**
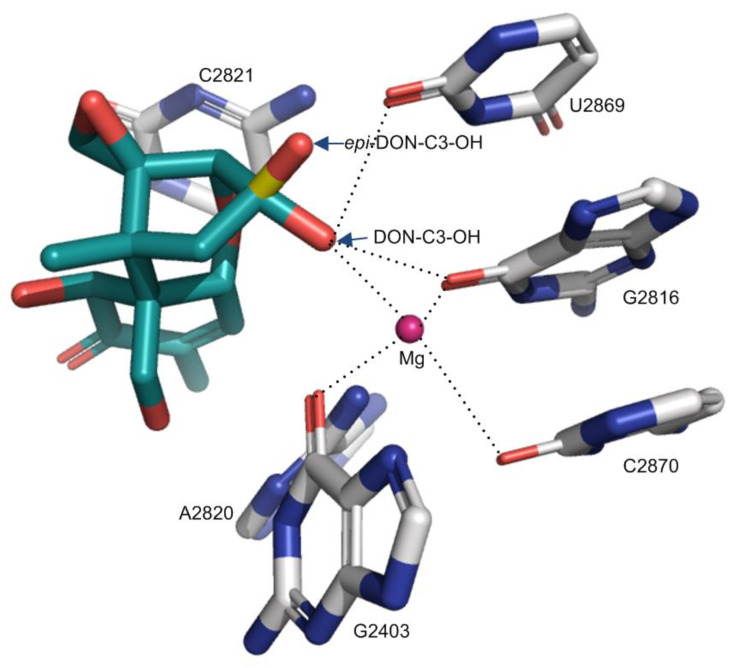
The effect of C^3^-OH conformation change in DON on the interactions to the ribosome. The C^3^-OH in 3-epi-DON was colored in yellow and red. The dotted lines indicate the binding interactions.

Although Pierron et al. (2016) have tried to rationalize these reduced toxicities through a structural analysis of trichothecene binding to ribosomes, the specific atoms participating in the interactions were not identified [24]. Based on the binding mode of trichothecenes reported in this study, the 3-epi-DON and 3-keto-DON will have the compromised binding contacts relative to DON (Figure 5, Table 1 and Table 2). Specifically, for 3-*epi*-DON, the distance between C^3^-O and Mg^2+^ will increase from 2.9 Å (DON) to 3.9 Å (3-epi-DON), weakening the interaction for metal ion coordination. Similarly, the distance between C^3^-O and O^2^-U2869 will slightly decrease from 2.7 Å (DON) to 2.3 Å (3-*epi*-DON). In summary, the binding contacts donated by epi-C^3^-OH will be compromised relative to DON while also likely introducing significant steric clashes (in the region of U2873 phosphate) (Figure 5). As for 3-keto-DON, the oxidation of C^3^-OH to the carbonyl group will lead to the loss of the hydrogen bond (as no donor will be present) and shift the position of the oxygen atoms so that its coordination to Mg^2+^ is compromised. Therefore, both 3-*epi*-DON and 3-keto-DON would be anticipated to have a weaker binding affinity to ribosomes relative to DON, with reduced toxicity (Table 2). In deepoxy-DON (DOM-1), the replacement of C^12,13^-O-epoxide by a hydrophobic -CH = CH2 group abolishes the hydrogen bond DON makes with the U2873 ribose 2′OH (Figure 2, Table 1). In addition, the loss of the epoxide oxygen reduces the bulk of this substituent, potentially entropically destabilizing the interaction. Therefore, the de-epoxidation of DON would be anticipated to weaken its binding to the ribosome. In summary, the oxidation and epimerization of C^3^-OH and de-epoxidation of 12,13-O-epoxide will impair the binding of trichothecenes to the ribosome, rationalizing the reduced toxicity observed for these transformed trichothecenes (Table 2) [24,39].

Intriguingly, the position at C^12,13^ epoxide ring and C^3^-OH have been primary targets for plants evolving resistance to FHB disease in general, and trichothecenes in particular. These modifications are exemplified by glucosyl conjugation at C^3^-OH [13] and glutathione substitution at C^13^ position [30]. Notably, the chemical conjugation of deoxynivalenol (DON) at C^13^ position with sulfur compounds was also identified [52].

Within the above-mentioned key structural elements to trichothecenes toxicities, the biological detoxification strategy targeting the double bond between C^9^ and C^10^ has not been reported to date. Reducing this bond would introduce significant conformational flexibility to the ring, with an out of a plane shift of the C16 methyl group that would compromise stacking interactions with A2820 and C2821. Therefore, potential biological detoxification strategies that target this position could hold promise for future trichothecene mitigation approaches.

## 4. Materials and Methods

### 4.1. Structure Analysis of Yeast 80S Ribosomes Complexed with Different Trichothecenes

The crystal structure data of yeast 80S ribosomes complexed with DON (PDB accession number: 4U53), T-2 toxin (PDB accession number: 4U6F), and verrucarin A (PDB accession number: 4U50) were used for the current analysis. The apo 80S ribosome (PDB accession number: 4V88) was also taken as the reference for the three-dimensional structure superimposition. Structure models were analyzed and visualized using PyMOL 2.3.4 (The PyMOL Molecular Graphics System. Schrödinger, Inc.; New York, NY, USA, 2020).

### 4.2. Relative IC_50_ Values of Different Trichothecenes

In order to compare the cytotoxicity of different trichothecenes, the toxicity data of selected trichothecenes was acquired from the previous reports (The relevant reference was list in Table 1) and converted into relative IC_50_ (the half-maximal inhibitory concentration) using equation 1. DON was set as the reference mycotoxin with a relative IC_50_ value of 1.0 using the same cell line model and experimental condition. The relative IC_50_ value greater than 1.0 indicated lower toxicity of the selected trichothecene than DON, while the lower relative IC_50_ value less than 1.0 indicated higher toxicity than DON under a specific test condition.

(1) $${\mathrm{Relative}\;\mathrm{IC}}_{50\;}=\frac{{\mathrm{IC}}_{50}\;\mathrm{of}\;\mathrm{DON}\;\mathrm{in}\;\mathrm{molar}\;\mathrm{concentration}\;}{{\mathrm{IC}}_{50}\;\mathrm{of}\;\mathrm{selected}\;\mathrm{trichothecene}\;\mathrm{in}\;\mathrm{molar}\;\mathrm{concentration}}\;$$

## 5. Conclusions

Thorough and in-depth comparative examination of the existing crystal structures of *Saccharomyces cerevisiae* 80S rRNA complexed with three trichothecenes (e.g., DON, T-2 toxin and verrucarin A), the key determinants for binding to 25S rRNA component of the 80S ribosome were identified. These interactions include metal ion coordination, hydrogen bonds and hydrophobic interactions. They are mainly contributed by the shared structural elements that are key to the toxicity of trichothecenes, including the oxygen in the C^12,13^-epoxide ring and a double bond between C^9^ and C^10^. Furthermore, the architecture of the trichothecene binding pocket in the ribosome was analyzed in relation to the substitution types in trichothecenes and their accommodation. The substitutions at different positions of trichothecenes impact their binding in two different patterns. The type I substitutions at C^4^, C^7^, C^8^, C^15,^ or C^16^ often introduce additional binding interactions and enhance trichothecene binding affinity to ribosome relative to their parent trichothecenes. In contrast, type II substitutions at C^3^, C^11^, or C^12^ positions often lead to the steric hindrance for the trichothecene’s binding to the ribosome and ultimately impair binding. Taking together, the structural basis of trichothecenes binding to the large subunit of ribosome presented in this study elucidated the mechanism behind the structure–activity relationship of trichothecenes. It explains why and how the key structural elements and substituents impact the toxic actions of trichothecenes specifically. This understanding further provides the scientific framework and guidance not only for the future development of strategies to detoxify trichothecenes in food and feed but also to improve the resistance of cereal crops to *Fusarium* fungal diseases.

## Figures and Tables

**Table 1:** Interactions between trichothecenes mycotoxins (DON, T-2 toxin and verrucarin A) and 80 S ribosome of yeast.

Trichothecenes	Atom in Trichothecenes (underlined)	Ribosome Residue/Metal Ion	Interaction Type	Distance(Å)	Comments
**DON Bound in 25S rRNA**					
DON	C^3^-OH	Mg^2+^	Metal ion coordination *	2.7–2.9	Binding contacts contributed by C^3^-OH
	C^3^-OH	O^2^-U2869	Hydrogen bond *	2.7–3.2	
	O-C^12,13^-epoxide	HO^2′^-β-ribose-U2873	Hydrogen bond *	2.5–2.8	Binding contacts formed by the core structure elements to the toxicity of trichothecenes
	C^6,7,8,9,10,11^ ring, along with C^16^ methyl	Cytosine ring in C2821	Hydrophobic stacking interaction	NA	
	C^6,7,8,9,10,11^ ring, along with C^16^ methyl	Adenine ring in A2820	Edge-to-edge hydrophobic interaction	NA	
T-2	C^3^-OH	Mg^2+^	Metal ion coordination	2.6	Binding contacts contributed by C^3^-OH
	C^3^-OH	O^2^-U2869	Hydrogen bond	2.9	
	O-C^12,13^-epoxide	HO^2′^-β-ribose-U2873	Hydrogen bond	2.7	Binding contacts formed by the core structure elements to the toxicity of trichothecenes
	C^6,7,8,9,10,11^ ring, along with C^16^ methyl	Cytosine ring in C2821	Hydrophobic stacking interaction	NA	
	C^6,7,8,9,10,11^ ring, along with C^16^ methyl	Adenine ring in A2820	Hydrophobic edge-to-edge interaction	NA	
	O-C^4^-OAc	HO^2′^-β-ribose-U2872	hydrogen bond	3.1	Additional contacts contributed by the substituents of T-2 toxin
	CH_3_-C^4^-OAc	G2403 and A2872	Hydrophobic interaction	NA	
	O^1^-C^15^-OAc	HN^2^-G2403	hydrogen bond	3.4	
	[OCOCH_2_CH(CH_3_)_2_]-C^8^-	A2820 and U2875	Hydrophobic interaction	NA	
Verrucarin A					
	O-C^12,13^-epoxide	HO^2′^-β-ribose-U2873	Hydrogen bond	2.8	Binding contacts formed by the core structure elements to the toxicity of trichothecenes
	C^6,7,8,9,10,11^ ring, along with C^16^ methyl	Cytosine ring in C2821	Hydrophobic stacking interaction	NA	
	C^6,7,8,9,10,11^ ring, along with C^16^ methyl	Adenine ring in A2820	Edge-to-edge hydrophobic interaction	NA	
	Additional ring between C^4^ and C^15^	ribose bases of A2872, U2873 and G2874	hydrophobic interaction	NA	Additional contacts contributed by the substituents of verrucarin A
	O-C^29^-ketone	HN^2^-G2403	Hydrogen bond	3.4	
	OH-C^28^	O^2^-U2875	Hydrogen bond	2.6	
	O-C^23^-ketone	HN^6^-A2401	Weak hydrogen bond	3.7	

The extended van der Waals contacts also contributed to the binding of trichothecenes to the ribosome. *: indicates the binding contacts mentioned in the previous publication [24]. The PDB accession number: 4U53 for deoxynivalenol, 4U6F for T-2 toxin, and 4U50 for verrucarin A. NA: not applicable.

## Data Availability

Not appliable.

## Abbreviations

FHB	*Fusarium* head blight disease
DON	Deoxynivalenol
DAS	Diacetoxyscirpenol
NIV	Nivalenol
OAc	Acetyl substituent
DON-3-Glc	Deoxynivalenol 3-glucoside
GSH	Glutathione
